# PROTAC EZH2 degrader-1 overcomes the resistance of podophyllotoxin derivatives in refractory small cell lung cancer with leptomeningeal metastasis

**DOI:** 10.1186/s12885-024-12244-3

**Published:** 2024-04-22

**Authors:** Min-xing Shi, Xi Ding, Liang Tang, Wei-jun Cao, Bo Su, Jie Zhang

**Affiliations:** 1 Department of Respiratory and Critical Care Medicine, Shanghai Pulmonary Hospital, School of Medicine, Tongji University, 200092 Shanghai, China; 2 Department of Tuberculosis, Shanghai Pulmonary Hospital, School of Medicine, Tongji University, 200092 Shanghai, China; 3 Department of Central Laboratory, Shanghai Pulmonary Hospital, School of Medicine, Tongji University, 200092 Shanghai, China; 4 Department of Oncology, Shanghai Pulmonary Hospital, School of Medicine, Tongji University, 200092 Shanghai, China

**Keywords:** Leptomeningeal metastases, Small cell lung cancer, Chemoresistance, Mouse model, PROTAC EZH2 degrader-1

## Abstract

**Background:**

Leptomeningeal metastasis (LM) of small cell lung cancer (SCLC) is a highly detrimental occurrence associated with severe neurological disorders, lacking effective treatment currently. Proteolysis-targeting chimeric molecules (PROTACs) may provide new therapeutic avenues for treatment of podophyllotoxin derivatives-resistant SCLC with LM, warranting further exploration.

**Methods:**

The SCLC cell line H128 expressing luciferase were mutated by MNNG to generate H128-Mut cell line. After subcutaneous inoculation of H128-Mut into nude mice, H128-LM and H128-BPM (brain parenchymal metastasis) cell lines were primarily cultured from LM and BPM tissues individually, and employed to in vitro drug testing. The SCLC-LM mouse model was established by inoculating H128-LM into nude mice *via* carotid artery and subjected to in vivo drug testing. RNA-seq and immunoblotting were conducted to uncover the molecular targets for LM.

**Results:**

The SCLC-LM mouse model was successfully established, confirmed by in vivo live imaging and histological examination. The upregulated genes included EZH2, SLC44A4, VEGFA, etc. in both BPM and LM cells, while SLC44A4 was particularly upregulated in LM cells. When combined with PROTAC EZH2 degrader-1, the drug sensitivity of cisplatin, etoposide (VP16), and teniposide (VM26) for H128-LM was significantly increased in vitro. The in vivo drug trials with SCLC-LM mouse model demonstrated that PROTAC EZH2 degrader-1 plus VM26 or cisplatin/ VP16 inhibited H128-LM tumour significantly compared to VM26 or cisplatin/ VP16 alone (*P* < 0.01).

**Conclusion:**

The SCLC-LM model effectively simulates the pathophysiological process of SCLC metastasis to the leptomeninges. PROTAC EZH2 degrader-1 overcomes chemoresistance in SCLC, suggesting its potential therapeutic value for SCLC LM.

**Supplementary Information:**

The online version contains supplementary material available at 10.1186/s12885-024-12244-3.

## Introduction

Lung cancer remains one of the most prevalent malignancies globally, accounting for approximately 2.2 million new cases and 1.8 million deaths annually [1]. SCLC, comprising about 15% of lung cancer cases, is notorious for its strong inclination toward distant metastasis and poor survival. In stark contrast to patients with non-small cell lung cancer (NSCLC), SCLC patients have not seen significant survival improvements over the past three decades. This stagnation is attributed to the absence of targeted therapies and the minimal efficacy of immunotherapy in treating SCLC [2]. Moreover, SCLC is particularly susceptible to recurrence and rapid development of drug resistance, leaving few alternative treatment options available [3].

Brain metastasis (BM) is still a major challenge in lung cancer treatment. For NSCLC patients with BM, survival have improved thanks to the introduction of novel drugs, including EGFR (NCT02616393) and ALK (NCT02336451) tyrosine receptor kinase inhibitors (TKIs) that can permeate the brain-blood barrier [4, 5]. However, chemotherapy has limited efficacy in improving the survival of SCLC patients with BM, especially in patients with chemoresistance. SCLC patients resistant to chemotherapy display substantially poorer overall survival (OS) compared to those responsive to chemotherapy [6]. Whole-brain radiotherapy, the main symptomatic control method for brain metastasis for the last 30 years [7, 8], has limited effectiveness in extending survival, primarily due to its toxic side effects [9].

Brain metastases include brain parenchymal metastasis (BPM) and leptomeningeal metastasis (LM). LM refers to the diffuse, multifocal and focal infiltration of malignant cells in the leptomeninges, subarachnoid space and other cerebrospinal fluid (CSF) compartments, which can disrupt the neuroaxis, leading to series of neurological symptoms [10, 11]. The overall incidence of LM is only 3-5% in solid tumours [10] but at least 10% in SCLC [12]. LM increases the intracranial pressure, resulting in intolerable cephalalgia, vertigo, nausea, and vomiting [13]. The prognosis for SCLC patients with LM is particularly grim, with a median survival of merely 6 weeks (range 2–22 weeks) [12, 14]. Traditional treatment of LM primarily focused on palliation, aiming to alleviating neurological symptoms and providing supportive services [15]. Besides standard therapies such as radiotherapy and systemic chemotherapy, intrathecal chemotherapy is also an option for patients with LM, but these treatment modalities do not significantly improve OS and are associated with an increased risk of side effects [16–19]. Thus, developing new treatments for refractory SCLC patients with LM is critically important.

In previous studies, EZH2 has been validated as a gene linked to drug resistance in SCLC, and inhibiting EZH2 has been shown to enhance the sensitivity of SCLC to chemotherapy [20, 21]. Proteolysis-targeting chimaeras (PROTACs) represent a novel drug development technology that utilize the ubiquitin‒proteasome system (UPS) to degrade target proteins [22]. PROTAC EZH2 degrader-1 [23] has been developed, and further research is needed to confirm its effectiveness in reversing drug resistance in SCLC.

In our study, a drug-resistant SCLC-LM mouse model was developed using MNNG-mutated H128 cells expressing luciferase. Drug testing in this mouse model demonstrated that PROTAC EZH2 degrader-1 could overcome chemoresistance in H128 cells, suggesting its potential as a therapeutic agent for treating refractory SCLC with LM.

## Methods

### Inducing cell mutations in vitro

NCI-H128 human SCLC cell line (ATCC, Chinese Academy of Sciences Cell Bank, Shanghai, China) was cultured in 90% RPMI 1640 medium, 10% foetal bovine serum, and 100 U/mL penicillin and streptomycin at 37 °C and 5% CO_2_. Approximately 4 × 10^5^ suspended H128 cells in 200 µL culture medium was transfected with CMV-Luc-PGK-Puro Lentivirus encoding firefly luciferase (Genomeditech, Shanghai, China) (multiplicity of infection, MOI = 20) by incubation at 37 °C for 30 min and then plated into culture dish. After 72 h, 5 µg/mL puromycin was used for screening to establish the H128-Luc cell line. For inducing mutations, N-methyl-N’-nitro-N-nitrosoguanidine (MNNG) (MedChemExpress, Shanghai, China) (20 µmol/L) was added into the culture medium of H128-Luc cells for 24 h to generate mutated H128 cells, and designated as H128-Mut.

### Screening cell clones in vivo

The H128-Mut cells (1 × 10^6^ per mouse) were seeded into the axillary region of 12 nude mice (6 weeks old, purchased from Shanghai Experimental Animal Center). Subsequently, the mice were monitored for the growth of subcutaneous tumours. The primary tumours were surgically excised after 6 weeks. After an 18-week period of inoculation, mice exhibiting intracranial metastases detected by in vivo live imaging were selected for further experiments. After anaesthesia, the tumour tissues from LM and BPM were separated for primary culture. Each mouse was euthanized by intraperitoneal injection of pentobarbital sodium at a dose of 130 mg/kg following the 2020 AVMA Guidelines for Animal Euthanasia.

The LM and BPM tumour tissues were promptly washed with PBS and minced into smaller pieces. After the addition of 0.125% trypsin, the tumour tissue was subjected to digestion at 37 °C for 15 min, with shaking once every 4 min. The resulting suspension was centrifugated, washed twice, and resuspended in RPMI 1640 medium. After passing through a 400-mesh sieve, single-cell suspension was obtained, and seeded into a 6-well plate. The cells were cultured at 37 °C in an incubator containing 5% CO_2_. Finally, primary H128-LM (leptomeningeal metastasis) and H128-BPM (brain parenchymal metastasis) cell lines were passaged for three successive generations (H128-LM1-3 and H128-BPM1-3, respectively).

### Biological characteristics of H128-BPM and H128-LM cells

#### Cell viability

Cells were cultured in 96-well plates at a concentration of 2 × 10^3^ cells/well. The incubation periods were set at 24, 48, 72, 96, and 120 h in a 5% CO_2_ incubator at 37 °C. Following each incubation period, 10 µL of CCK8 solution was introduced into each well and incubated for an additional 30 min. The absorbance at 450 nm was determined to evaluate cell viability.

#### Transwell assay

Transwell experiments were performed in 24-well plates. Two hundred microlitres of serum-free cell suspension was added to each upper chamber at a cell density of 2.5 × 10^5^ cells/mL, and 10% FBS complete medium was added to the lower chamber. After 24 h, the cells in the lower chamber were counted and photographed.

#### Pathology and histopathology

Tumour tissues were dehydrated, embedded in paraffin and sectioned at 4 µm. The slices were stained with haematoxylin and eosin (H&E) after dewaxing and hydration The slices were subjected to antigen retrieval at 96°C for 40 minutes. Endogenous peroxidase activity was blocked with 3% hydrogen peroxide. The slices were incubated overnight with primary antibodies against SYP (GB11553), CHGA (GB111316), CD56 (GB112671) (Servicebio, Wuhan, China) or INSM1 (ab305104, Abcam, USA). Then, the slices were incubated with goat anti-rabbit HRP-linked IgG (LF102 Epizyme Biomedical Technology, Shanghai, China) or anti-mouse IgG HRP secondary antibody (LF101 Epizyme Biomedical Technology, Shanghai, China), washed and stained with 3,3’-deaminobenzidine (DAB). Immunohistochemical staining was graded as follows: -, negative; +, weakly positive; ++, positive; and +++, strongly positive.

### Establishment of a mouse model of SCLC leptomeningeal metastasis

#### Mouse model of SCLC with leptomeningeal metastasis

The animal study was approved by the ethics committee of Shanghai Pulmonary Hospital, Animal Care and Use Committee of Tongji University. Six-week-old nude mice were purchased from Shanghai Experimental Animal Centre. The feeding and experimental model was conducted in accordance with the Institutional Animal Care and Use Committee (IACUC) guidelines. After anaesthesia with 50 mg/kg pentobarbital sodium, 1 × 10^6^ H128-LM cells were inoculated into the right carotid artery of each nude mouse, and the incision was closed by ligation with silk thread. On the 7th, 21st and 35th days after inoculation, the growth of intracranial tumours was monitored by in vivo imaging, and the mice were euthanized on the 35th day.

#### In vivo bioluminescence imaging (BLI)

After isoflurane anaesthesia, the mice were placed in the Tanon 6600 imaging system, and scanning began 5 min after the intraperitoneal injection with D-luciferin (150 mg/kg), lasting for 15 min. The regions of interest (ROIs) were drawn, including the tumour, and the fluorescence intensity of the ROIs was quantified by Tanon 6600 imaging software.

#### RNA sequencing

To compare the gene expression profiles of H128 cells, H128-BPM cells, and H128-LM cells, RNA sequencing (RNA-seq) was performed on total RNA extracted using TRIzol reagent (Invitrogen). RNA-seq reads were mapped to the human reference genome with STAR v.2.6.1b. Differential expression analysis of genes was performed with the R package DESeq2.

#### Western blotting

The total proteins of H128-LM and H128-BPM cells were extracted and subjected to 10% SDS‒PAGE, transferred onto nitrocellulose (NC) membranes and hybridized with the following antibodies: CDH1 (24E10, CST, USA), β-ACTIN (ab8227, Abcam, USA), CDH2 (A19083, ABclonal, Shanghai, China), MMP9 (A2095 ABclonal, Shanghai, China), EZH2 (A19577 ABclonal, Shanghai, China), VEGFA (A12303 ABclonal, Shanghai, China), SLC44A4 (A10435 ABclonal, Shanghai, China), OCLN (A2601 ABclonal, Shanghai, China), IGF2 (A2086 ABclonal, Shanghai, China). Goat anti-rabbit HRP-linked IgG (LF102 Epizyme Biomedical Technology, Shanghai, China) or anti-mouse IgG HRP were used as secondary antibodies (LF101 Epizyme Biomedical Technology, Shanghai, China). The protein bands were visualized using enhanced chemiluminescence (ECL) in ChemiDoc Imaging System (Bio-Rad).

### Drug efficacy evaluation

#### Cytotoxicity assay

To assess the efficacy of the EZH2 inhibitor PROTAC EZH2 degrader-1 in reversing resistance in vitro, four different drugs were employed alone and in combination with PROTAC EZH2 degrader-1 (MedChemExpress, Shanghai, China): carboplatin (CBP), etoposide (VP16), teniposide (VM26) (MedChemExpress, Shanghai, China), and anlotinib (CHIATAI TIANQING, Shanghai, China). Four kinds of cells (8 × 10^3^ cells/well), H128, H128-Mut, H128-BPM, and H128-LM, were seeded in 96-well plates and treated with serially diluted drugs for 72 h.

After the addition of CCK-8 for half an hour, the absorbance at 450 nm was measured to calculate relative cell viability as the ratio of absorbance in treated cells to control cells.

#### In vivo drug testing

**Fig. 1:**
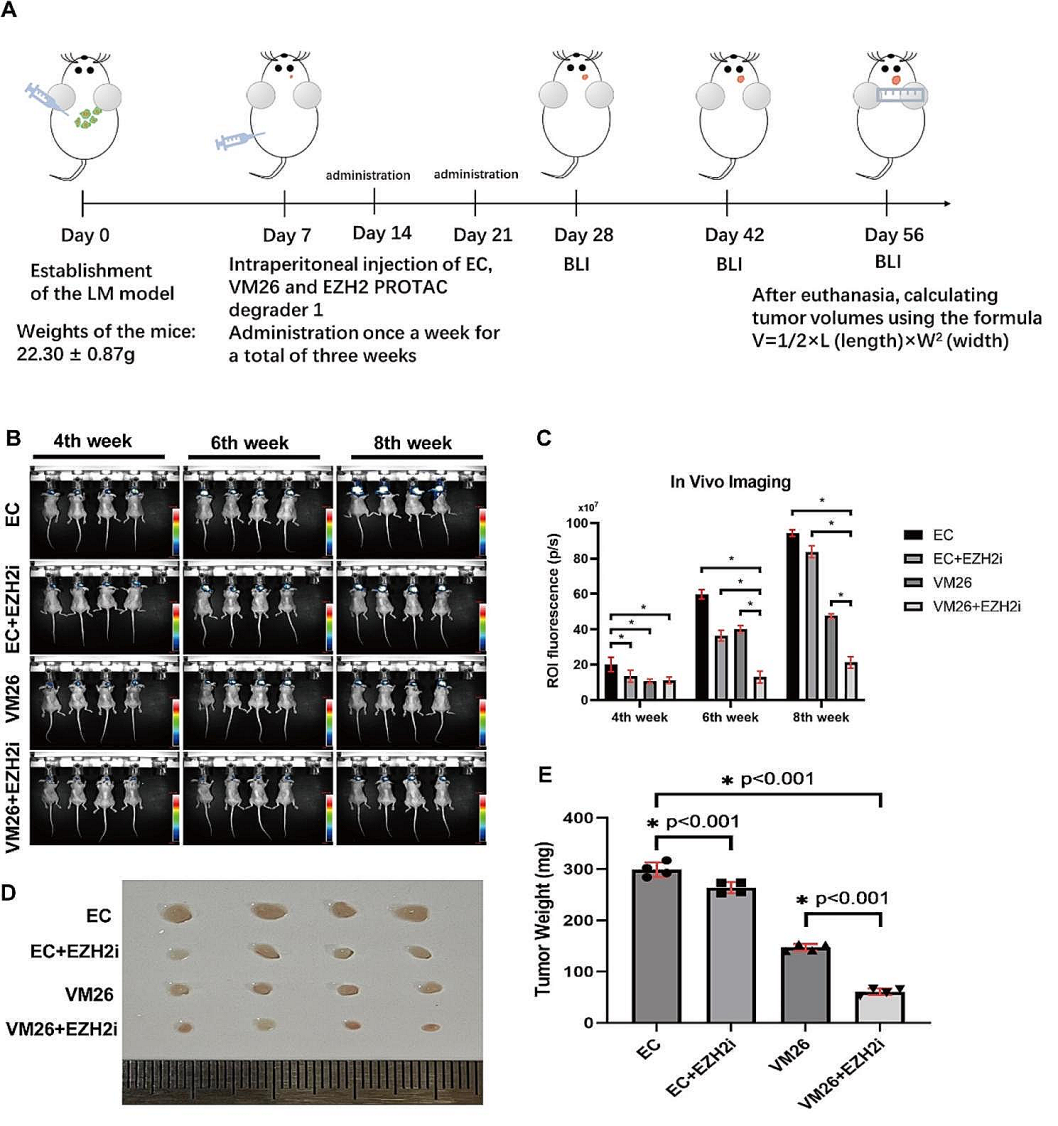
**A**: Timeline of in vivo drug testing in this study. **B**: In vivo imaging of leptomeningeal metastases in mice. The colour scale indicates the photon flux (photon/s) emitted from each group. **C**: Quantitative bar graph representing the total photon flux calculated from the region of interest. The data are presented as the means ± SDs, “*” indicates *P* < 0.05. **D**: Following the euthanasia of the mice, LM tumour tissues were extracted and subjected to weight measurements. **E**: The mean tumour weight is depicted in the bar graphs; error bars represent standard deviation. “*” represents *P* < 0.05

One week after establishment of the LM model, 16 mice were divided into four groups (VP16 + CBP group, EC; VP16 + CBP + EZH2 inhibitor group, EC + EZH2i; VM26 group, VM26; and VM26 + EZH2 inhibitor group, VM26 + EZH2i). LM model mice received drug treatments according to the different groups. EC was administered intraperitoneally on Days 2 of each cycle (VP16: 4 mg/kg, CBP: 80 mg/kg); VM26 was administered on Day 2 of each cycle (2.5 mg/kg); and EZH2i was administered on Day 1 of each cycle (0.5 mg/kg). All treatments were repeated in 3 cycles, with 7 days per cycle. After three cycles of treatment (3 weeks), in vivo live imaging was conducted biweekly to track the progression of intracranial tumours. At the end of the 8th week, the mice were euthanized, and intracranial tumour tissue was extracted (Fig. 1A).

### Statistical methods

Immunohistochemical scoring data analysis was performed using the Kruskal-Wallis test, while RNA-seq data analysis employed the DESeq2 package in the R programming language. A curve fitting method was applied to estimate the half-maximal inhibitory concentration (IC50) values from in vitro drug experiments, and one-way analysis of variance (ANOVA) was utilized for statistical analysis of Transwell assays, weights of tumours and fluorescence intensities.

## Results

### H128-LM cells were established from SCLC leptomeningeal metastases in mice

**Fig. 2:**
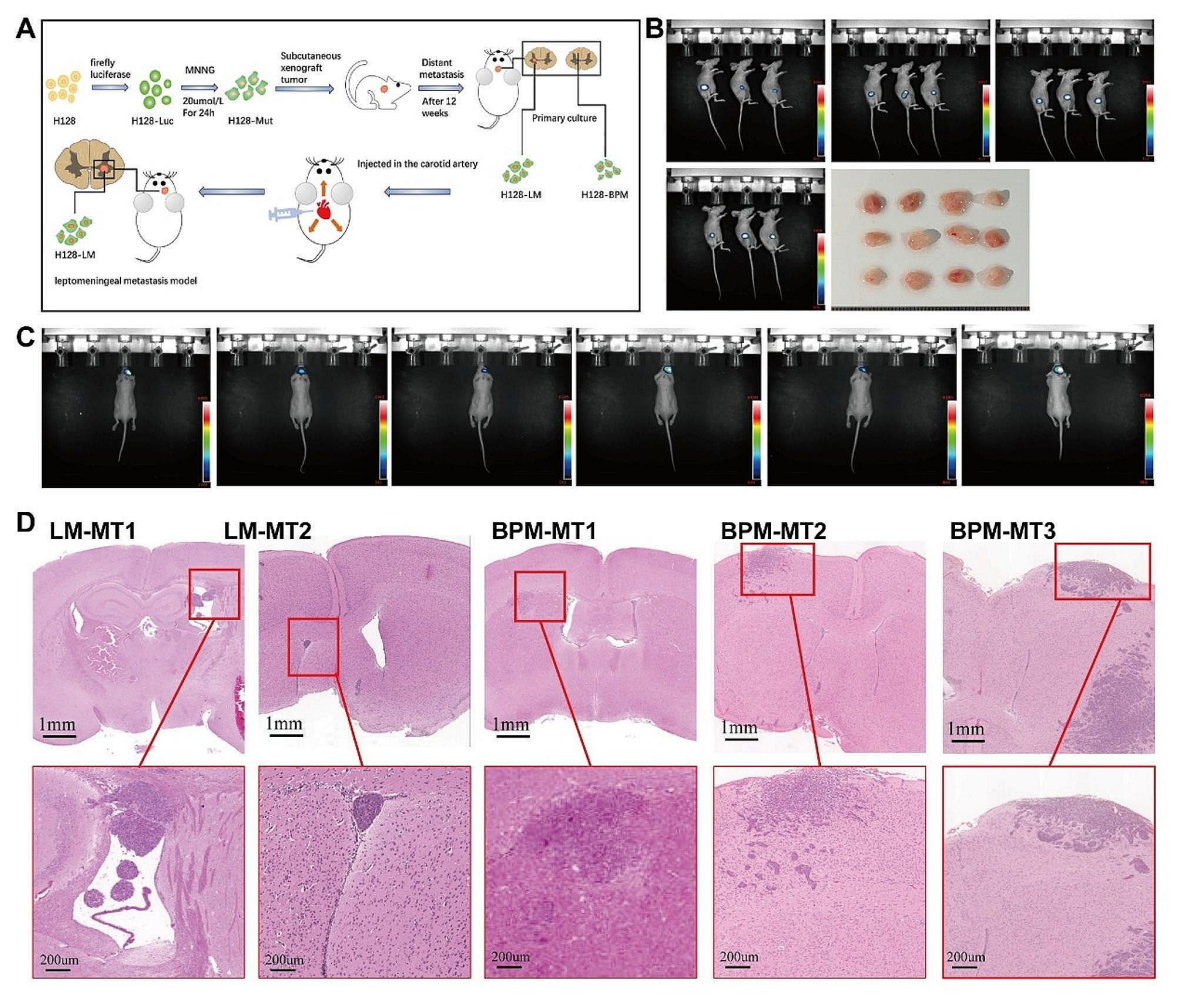
**A**: Study flow chart. **B**: Live imaging of 12 mice subcutaneously transplanted with = tumour cells and subcutaneous tumour tissue extracted after resection. **C**: 5 live brain tumour metastasis images. **D**: Mouse brain tissue sections subjected to H&E staining

H128-Mut cells were generated by transfecting a firefly luciferase expression vector with mutated by MNNG (as detailed in Methods). The H128-Mut cells were subcutaneously inoculated into nude mice, and LM lesions were removed after 12 weeks and histological examination confirmed the presence of LM. The H128-LM cell line primarily cultured from LM tumour tissue. Subsequently, H128-LM cells were inoculated into nude mice *via* the carotid artery, and intracranial occupancy was detected using in vivo live imaging (Fig. 2A).

H128-Mut cells were injected subcutaneously into 12 mice. After 4 weeks, tumour resection was performed at the inoculation site, with the mean tumour diameter being 0.9 ± 0.37 cm. (Fig. 2B). By the 12th week, intracranial metastasis was observed in 6 mice by in vivo live imaging (Fig. 2C), followed by brain tissue sectioning and H&E stained (Fig. 2D). The tumour cells were characterized as small, oval-shaped cells, with minimal cytoplasm and positive immunohistochemical staining of neuroendocrine markers. Microscopic examination of tissue sections showed that tumours invading the ventricles and pia mater were identified as LM (Fig. 2D LM-MT1 and LM-MT2), whereas tumours invading the brain parenchyma were classified as BPM (Fig. 2D BPM-MT1, BPM-MT2, and BPM-MT3). Histopathological findings were independently determined by 2 pathologists. Two cell lines (H128-LM and H128-BPM) were established by primarily culturing LM tumour tissues and BPM tumour tissues. The cells were passaged for three generations, resulting in the creation of H128-LM1-3 and H128-BPM1-3 cell lines.

Furthermore, at the endpoint of in vivo experiments, most mice with BPM exhibited symptoms like decreased appetite and bradykinesia. Mice with LM displayed more severe symptoms, including anorexia, bradykinesia, and even astasia, closely mirroring some clinical symptoms in humans.

### The biological characteristics of H128-LM and H128-BPM cells

**Fig. 3:**
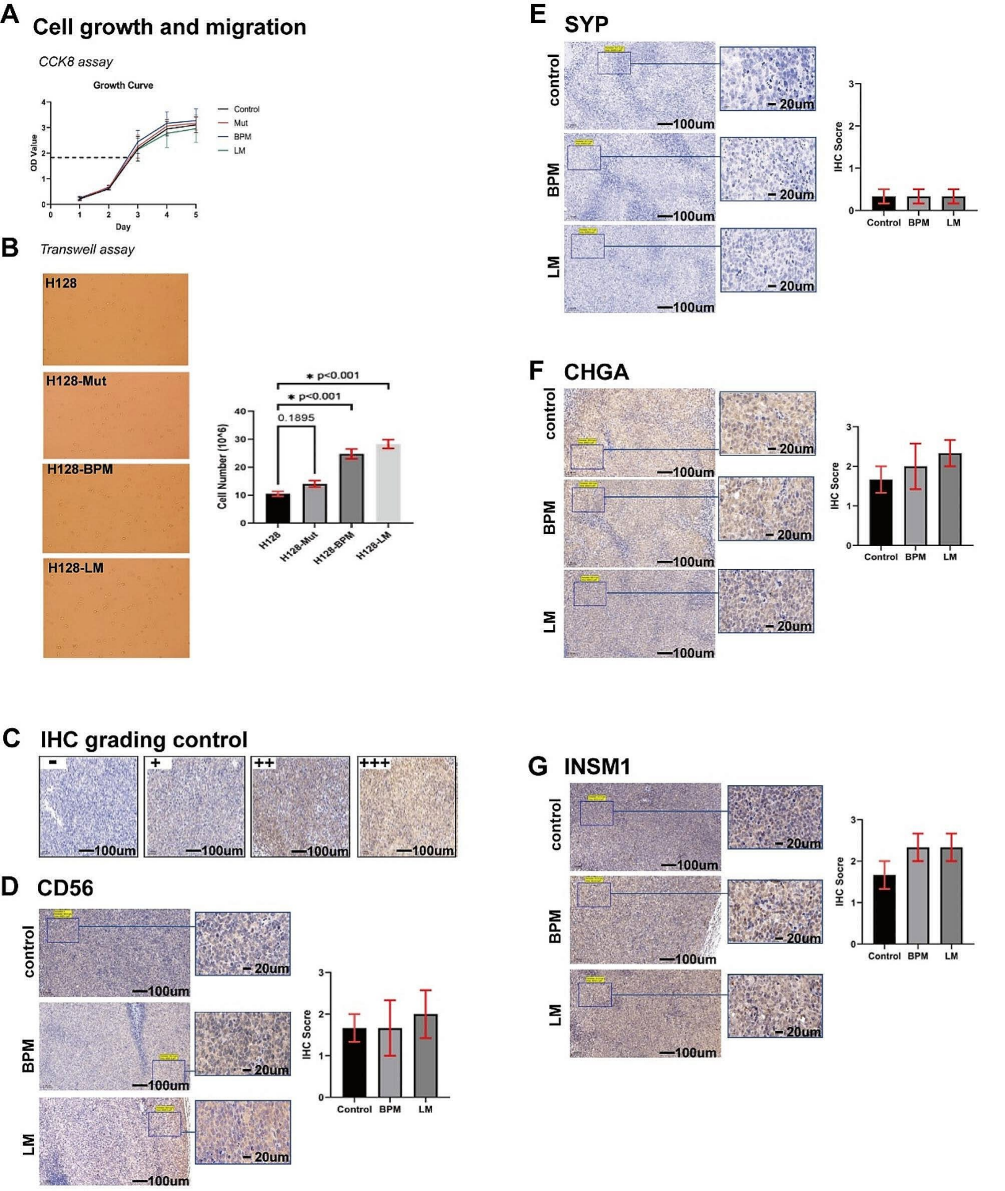
**A**: A CCK-8 assay was used to determine tumour cell growth. **B**: Transwell assay; tumour cells in the lower chamber were viewed at 10× magnification, “*” indicates *P* < 0.05. **C**: Immunohistochemical grading control **D-G**: Immunohistochemistry of subcutaneous tumour tissue (control), brain parenchyma metastatic tissue, and leptomeningeal metastatic tissue at 10x objective and 63x objective, respectively: (**D**) CD56 primary antibody (*P* = 0.88); (**E**) SYP primary antibody; (**F**) CHGA primary antibody (*P* = 0.58); (**G**) INSM1 primary antibody (*P* = 0.33)

We compared the growth and migration capabilities of the four cell lines, H128, H128-Mut, H128-LM, and H128-BPM (Fig. 3A&B). We found that though there was little difference between the growth of the four kinds of cells, the migration ability of H128-LM and H128-BPM was significantly higher compared to the original H128 and H128-Mut cells (*P* < 0.001).

Subcutaneous transplant tumour tissues (H128-Mut, control), BPM tumour tissues, and LM tumour tissues were subjected to IHC analysis, which showed that H128-LM and H128-BPM still have the expression of neuroendocrine markers, including CD56, CHGA, SYP and INSM1 (Fig. 3C-G, *P* > 0.05). This suggested that the pathological type of original H128 did not change after selection and transplantation.

It is demonstrated that though the proliferative ability has little difference between the cells, the intracranial metastatic cells have stronger migration ability.

### High expression of EZH2 and SLC44A4 in H128-LM cells

**Fig. 4:**
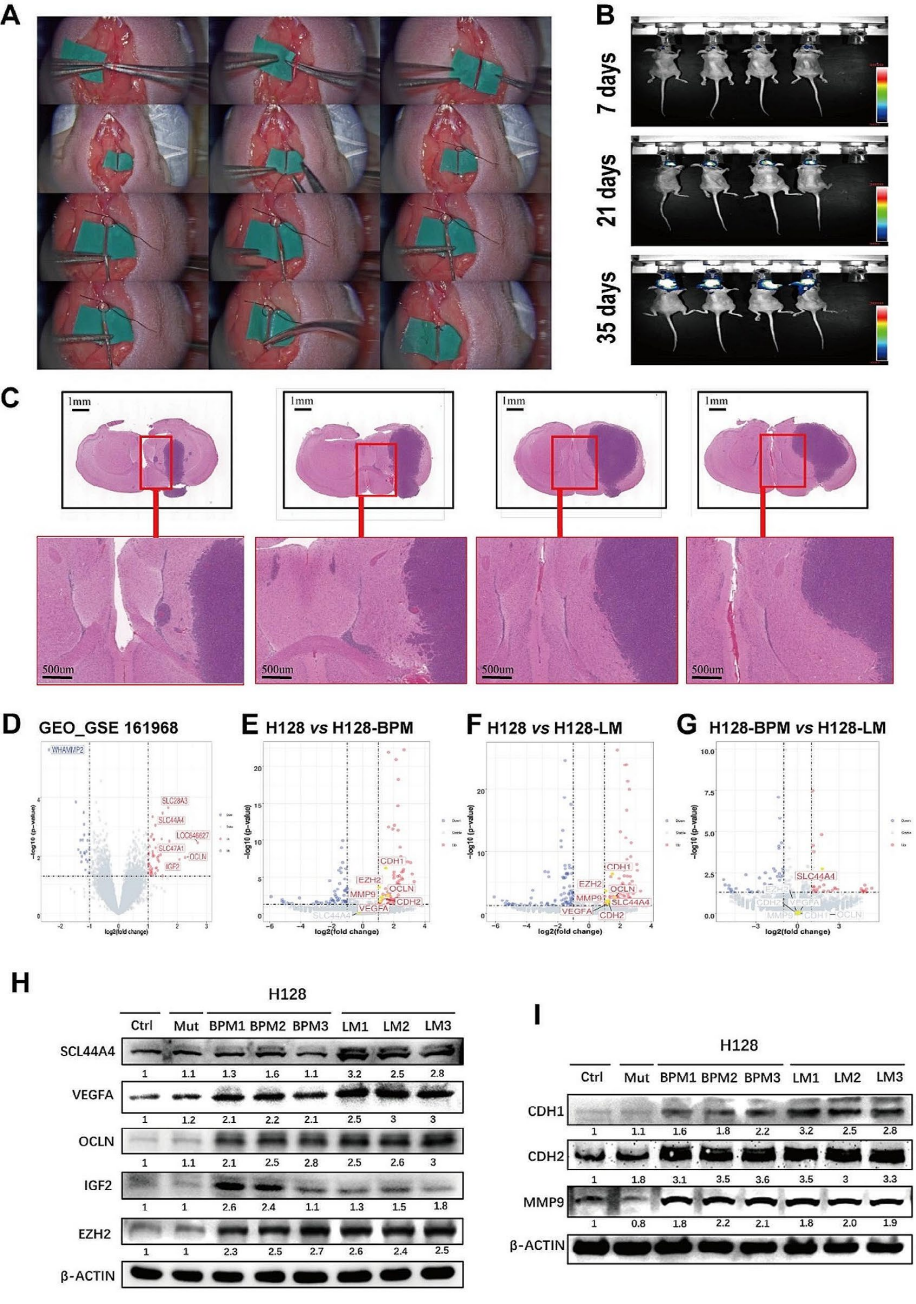
**A**: H128-LM cells were injected via the right carotid artery. **B**: In vivo imaging of leptomeningeal metastasis in mice. **C**: Leptomeningeal metastasis tumour tissue, H&E staining. **D-G**: Volcano plot demonstrating differential gene expression, especially genes with upregulated expression, with a twofold threshold and *P* value < 0.05. (**D**): GEO database; (**E**): H128 cells and H128-BPM cells; (**F**): H128 cells and H128-LM cells; (**G**): H128-BPM cells and H128-LM cells. In Panels E, F, and G, there were 2 samples per group. **H, I**: The H128-LM and H128-BPM cell lines were passaged for three generations. Immunoblot analysis of SCL44A4, VEGFA, OCLN, IGF2, EZH2, CDH1, CDH2 and MMP9 expression in four H128 cell lines (H128, H128-Mut, H128-LM, H128-BPM); beta-actin was used as a loading control

H128-LM cells were injected into the right carotid artery of the nude mice (Fig. 4A). In vivo live imaging was performed on Days 7, 21 and 35 (Fig. 4B). Following these procedures, the mice were euthanized, and brain with LM tumour was collected for H&E staining (Fig. 4C). Histological examination confirmed the infiltration of tumour cells into the leptomeninges.

To investigate the differences in gene expression between H128-LM cells and other cell types, we initially analysed RNA-seq data from SCLC brain metastases available in the Gene Expression Omnibus (GEO) database (Series GSE 161,968) and found that SLC44A4, OCLN, IGF2, SLC47A1, and SLC28A3 were significantly upregulated in patients with brain metastases (Fig. 4D). Further, to assess the gene expression profiles of the H128, H128-LM, and H128-BPM cell lines, RNA-seq was performed. The SLC44A4 gene exhibited a significantly upregulated expression in H128-LM cells compared to the other two cell lines (Fig. 4E-G). Notably, EZH2, known to be associated with drug resistance, demonstrated a significant upregulation in H128-LM and H128-BPM cells in comparison to H128 cells (Fig. 4E-G). Additionally, higher expression of IGF2 and OCLN were observed in both H128-LM and H128-BPM cells compared to that of H128 cells (Fig. 4E-G). Furthermore, the expression of VEGFA, a ligand for VEGFR, was increased in both H128-LM and H128-BPM cell lines when compared to the H128 control group (Fig. 4E-G). The genes CDH1, CDH2, and MMP9, which are involved in promoting tumour cell metastasis [24], was significantly increased in H128-LM cells and H128-BPM cells compared to H128 cells (Fig. 4E-G).

To validate the RNA-seq results, we performed Western blotting among the H128, H128-Mut, H128-BPM, and H128-LM cell lines (Fig. 4H, I). The expression of SLC44A4 was higher in H128-LM cells compared to the others (Fig. 4H). Additionally, the upregulated expression of EZH2 was observed in H128-LM and H128-BPM cells compared to H128 and H128-Mut cells (Fig. 4H). The expression of EMT-associated genes CDH1, CDH2, and MMP9 was significantly increased in H128-LM and H128-BPM cells (Fig. 4I).

### PROTAC EZH2 degrader-1 significantly enhanced the sensitivity of SCLC leptomeningeal metastases to chemotherapy in vitro and in vivo

#### In vitro drug testing

**Fig. 5:**
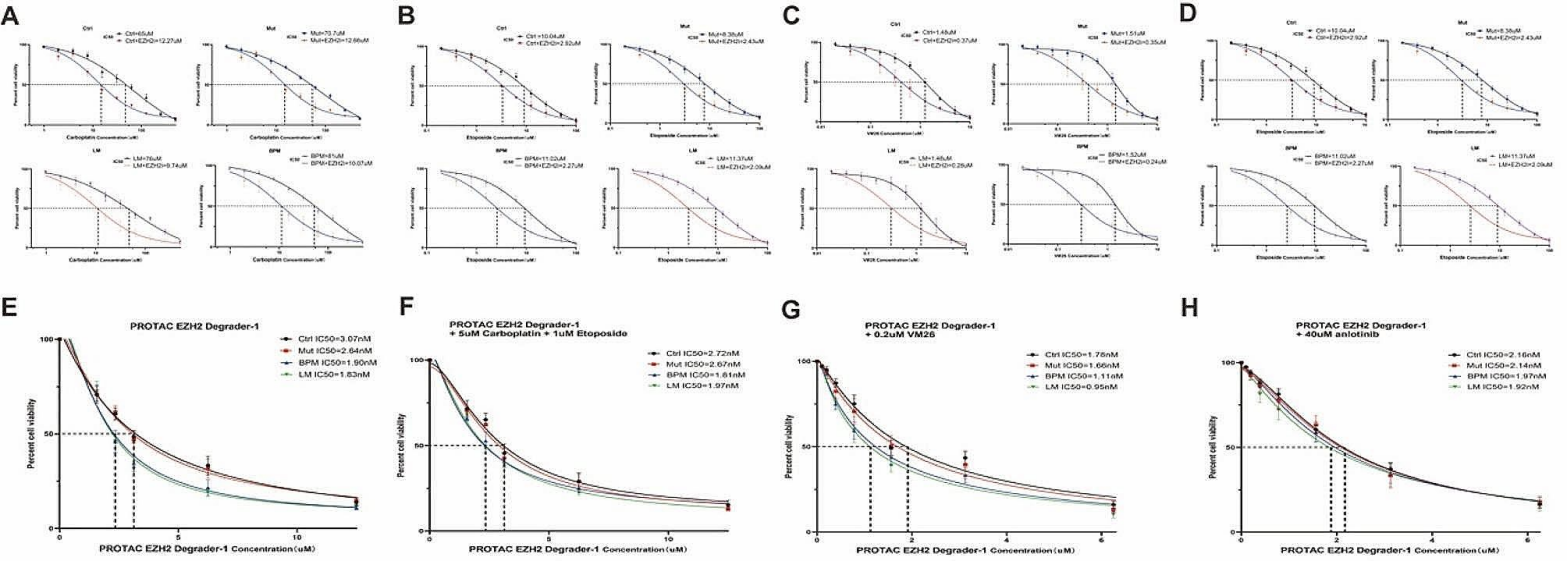
**A**-**C**: The sensitivity of H128, H128-Mut, H128-BPM or H128-LM cells to carboplatin (**A**), etoposide (**B**) and teniposide (**C**) through the EZH2 degrader. H128 cells were pretreated with PROTAC EZH2 degrader-1 (2 nM) followed by carboplatin (**A**), etoposide (**B**) or teniposide (**C**) treatment for 72 h prior to cell viability assays. **D**: The viability of cells treated with anlotinib, both alone and in combination with the EZH2i regimen, was examined. **E-H**: Cell viability was measured 72 h after treatment with PROTAC EZH2 degrader-1 alone (**E**) or in combination with three regimens (F: carboplatin + etoposide, G: VM26, H: anlotinib)

All the four H128 cell lines were utilized for drug sensitivity testing using CCK8 assay. The IC50 values for carboplatin, VP16 and VM26 were significantly decreased in all cells treated with PROTAC EZH2 degrader-1 (Fig. 5A-C). In H128-LM cells, the IC50 values for carboplatin, VP16, and VM26 were 76 µM, 11.37 µM, and 1.48 µM individually (Fig. 5A-C). Upon combination with the PROTAC EZH2 degrader-1, these values decreased to 9.74 µM, 2.09 µM, and 0.28 µM, respectively (*P* < 0.05) (Fig. 5A-C). The H128-LM cells exhibited the greatest sensitivity (IC50 = 1.97 nM) to the PROTAC EZH2 degrader-1 when treated with carboplatin and etoposide (Fig. 5F), and a similar trend was observed in VM26 (Fig. 5H). The small molecule inhibitor anlotinib did not show a significant decrease in its IC50 value after combination with PROTAC EZH2 degrader-1 (Fig. 5D and H).

We also found that the IC50 values of PROTAC EZH2 degrader-1 were lower in H128-LM (1.83 nM) and H128-BPM cells (1.90 nM) compared to H128 cells (3.07 nM, *P* < 0.05) and H128-Mut cells (2.64 nM, *P* < 0.05) (Fig. 5E). This result could be attributed to the upregulation of EZH2 expression in both cell lines (Fig. 4H). Among the three coupling schemes examined, the combination of PROTAC EZH2 degrader-1 with VM26 demonstrated the most advantageous outcome, as evidenced by the low IC50 value, particularly for H128-LM cells (IC50 = 0.95 nM) (Fig. 5G).

#### In vivo drug testing

After establishment of the LM models, the drugs were administered with three treatment cycles (Fig. 1A). We observed that the intracranial tumours in the EZH2i group showed a decrease in luminous intensity compared to the control group by in vivo imaging (Fig. 1B). Fluorescence intensity analysis revealed that VM26 + EZH2i inhibited intracranial tumour growth more effectively than EP + EZH2i (Fig. 1B). VM26 + EZH2i was the most effective combination among the tested four kinds of drug combinations (Fig. 1C).

The administration of the EZH inhibitor inhibited intracranial tumour growth (Fig. 1C). The tumour weight decreased significantly (*P* < 0.001) (Fig. 1E). This effect was significant after the administration of the EZH inhibitor (Fig. 1D). The mean tumour weight in the EP + EZH2i group was significantly lower compared to the EP group (263.7 ± 11.0 mg vs. 299.0 ± 14.2 mg, *P* < 0.01). Similarly, the mean tumour weight in the VM26 + EZH2i group was significantly lower compared to the VM26 group (60.64 ± 6.5 mg vs. 147 ± 6.7 mg, *P* < 0.01). The most pronounced tumour inhibitory effect was observed in the VM26 + EZHi group (Fig. 1E).

## Discussion

The challenge in treating SCLC brain metastases, particularly leptomeningeal metastasis, is significantly compounded by the presence of the blood‒brain barrier (BBB) [25]. Radiotherapy can increase the permeability of BBB, facilitating chemotherapeutic drugs’ access to the brain [26]. However, the efficacy of whole-brain radiation therapy in treating leptomeningeal metastasis is minimal [15]. The treatment of drug-resistant SCLC patients with LM is even less promising. For other refractory tumours, such as triple-negative breast cancer, chemoresistant cell lines [27] and animal models for intracranial and bone metastasis have been developed [28]. Therefore it is necessary to establish preclinical animal and cell models for the study of refractory SLCL with intracranial metastasis [8], particularly LM.

In this study, a chemoresistant SCLC cell line H128 was subjected to in vivo selection, resulting in the H128-LM cell line from primary culture of LM tumour tissue. To establish LM mouse model, the H128-LM cells were inoculated into the carotid artery of nude mice. Three weeks after inoculation, the formation of intracranial metastases was observed by BLI. Compared to contrast-enhanced resonance imaging (CE-MRI), BLI can simultaneously scan multiple mice, reducing the potential for errors in efficacy experiments between groups. Additionally, D-luciferin, which is injected for in vivo imaging, does not affect tumour growth and is non-toxic [29], while the use of gadolinium-based contrast agents in CE-MRI may pose potential risks [30]. Compared with the previously reported LM models using intraventricular [31] and intrathecal injection [32–35], our LM model more accurately simulated pathological process of leptomeningeal metastasis while also minimizing harm to animals.

Both H128-LM cells and H128-BPM cells retained the neuroendocrine characteristics unique to SCLC by showing high expression of CD56, CHGA and INSM1, which indicates that LM cells and BPM cells retain the biological characteristics of SCLC. In this study, the findings of the Transwell experiments showed that H128-LM cells and H128-BPM cells exhibit greater invasiveness compared to the original H128 cells. This enhanced migration ability might be attributed to the upregulation of EMT-related genes (CDH1, CDH2, MMP9, and OCLN) in both the H128-LM and H128-BPM cells. The result from the RNA-seq and western blot assays also showed that the higher expression of VEGFA in H128-BPM cells and H128-LM cells compared to H128 cells and H128-Mut cells, which indicates that the ability to promote angiogenesis in intracranial metastases cells is enhanced.

EZH2 is the catalytic subunit of polycomb repressive complex 2 (PRC2) [36], which has histone methyltransferase activity and can catalyse the methylation of lysine 27 of histone H3 (H3K27) to regulate the expression of tumour suppressor genes [37]. EZH2 is highly expressed in H128 cells, a drug-resistant SCLC cell line [20, 38].. High expression of EZH2 in H128 cells may contribute to drug resistance. Our research not only verified that EZH2 is highly expressed in H128 cells but also showed that EZH2 is highly expressed in H128-BPM and H128-LM cells (Fig. 1E). Previous studies have shown that EZH2 plays a crucial role in tumour resistance to chemotherapy including SCLC [39], breast cancer [40], ovarian cancer [41, 42], and head and neck cancer [43]. Koyen et al. [21] reported that when EZH2 was knocked down in H128 cells, the mechanism of drug resistance in SCLC could be reversed. To date, two kinds of EZH2 inhibitors have been investigated in clinical research for the treatment of drug-resistant tumours. The EZH2 inhibitor Valemetostat [44] had an objective response rate (ORR) of 48% in a phase 2 clinical trial (NCT04102150) in the treatment of patients with relapsed or refractory (R/R) adult T-cell leukaemia/lymphoma (ALT). The other EZH2 inhibitor Tazemetostat [45] also showed excellent results in a phase II clinical trial (NCT01897571) for the treatment of relapsed or refractory follicular lymphoma. In the EZH2 mutation cohort, the ORR of Tazemetostat was 69%, while that in the EZH2 wild-type cohort was only 35%. These studies demonstrate that EZH2 is a key gene in tumour resistance.

Moreover, EZH2 plays a crucial role in multiple immune processes [46]. It facilitates TCR stimulation and promotes interactions between T cells and antigen-presenting cells, thereby enhancing T-cell activation [47]. EZH2 is also essential for the differentiation and activation of Treg cells [48–50]. EZH2 regulates the activation, proliferation, and differentiation of CD8 + T cells through histone methylations [50]. Additionally, EZH2 is involved in the differentiation of TH1, TH2, and TH17 cells [49]. Nevertheless, the immunodeficient mice model we employed in this study can only assess the direct inhibition of PROTAC EZH2 Degrader-1 on tumour cells. Currently, in several preclinical model studies, EZH2 inhibitors have been shown to enhance the effectiveness of immune checkpoint blockade (ICB) [51, 52] and have demonstrated the ability to overcome acquired resistance to ICB [53]. The evaluation of EZH2 regulation on the immune response to tumours needs immune proficient mouse model or humanized immune system mouse models. Future research is needed to elucidate the immunological role of PROTAC EZH2 degrader-1 in treatment of SCLC.

Recently, PROTACs [22] have been developed for inhibiting EZH2 [37, 54, 55]. PROTACs are advantageous due to their high activity, low toxicity, broad target specificity, and ability to overcome challenges posed by target gene mutations [56]. After binding to the ligand, PROTACs enter the tumour cell, bind specifically to one end of the EZH2 protein, and recruit ubiquitin E3 ligase at the other end to form a triplet EZH2-PROTAC-E3 ligase complex [22]. This leads to EZH2 degradation by the ubiquitin‒proteasome system [18]. We found that the IC50 values of VP-16, carboplatin and VM26 in tumour cells treated with PROTAC EZH2 degrader-1 were significantly lower than those in tumour cells without PROTAC EZH2 degrader-1. In vivo, experiments showed that combining chemotherapy with PROTAC EZH2 degrader-1 more effectively controlled the growth of leptomeningeal metastatic tumours in nude mice than chemotherapy alone. Our results indicated that PROTAC EZH2 degrader-1 could overcome resistance by inhibiting EZH2 and suppressing tumour growth, which contradicts previous reports showing that EZH2 is responsible for drug resistance in SCLC [20, 21, 40]. However, we found that PROTAC EZH2 degrader-1 did not improve the sensitivity of SCLC to the small molecule multitargeted anlotinib, showing that PROTAC EZH2 degrader-1 could only overcome the resistance of H128 cells to chemotherapeutic drugs. Notably, PROTEC EZH2 degrader-1 showed significant therapeutic efficacy in treating refractory SCLC leptomeningeal metastasis in our in vivo experiments. However, further clinical research is still needed to validate these findings.

Our RNA-seq analysis of LM cells revealed that SLC44A4 was specifically expressed in H128-LM cells, aligning with data from the GEO database on SCLC brain metastases. SLC44A4 is a member of the SLC44 choline transporter family, which is involved in choline synthesis [57]. Song et al. [58] verified that SLC44A4 could promote cell proliferation by regulating acetylcholine secretion in SCLC cells. In our study, the significant upregulation of SLC44A4 protein expression in H128 leptomeningeal metastatic cells was validated through western blotting. Based on these results, we hypothesize that the SLC44A4, uniquely expressed in tumour cells metastasizing to the leptomeninges, could serve as a potential therapeutic target for SCLC leptomeningeal metastasis.

In conclusion, our study demonstrates that the PROTAC EZH2 degrader-1 can penetrate the blood-brain barrier and overcome the resistance to podophyllotoxin derivatives (VM26) in the refractory SCLC-LM model, suggesting a potential therapeutic strategy for treating SCLC leptomeningeal metastasis.

### Electronic supplementary material

Below is the link to the electronic supplementary material.

Supplementary Material 1

## Data Availability

The datasets generated and/or analysed during the current study are available in 10.6084/m9.figshare.24901683.v1.
