# Supplementary figures and images for: PROTAC EZH2 degrader-1 overcomes the resistance of podophyllotoxin derivatives in refractory small cell lung cancer with leptomeningeal metastasis
Source: BMC Cancer. 2024 Apr 22;24:504. doi: 10.1186/s12885-024-12244-3 (PMC11034131; doi:10.1186/s12885-024-12244-3)

**Figure3 H**


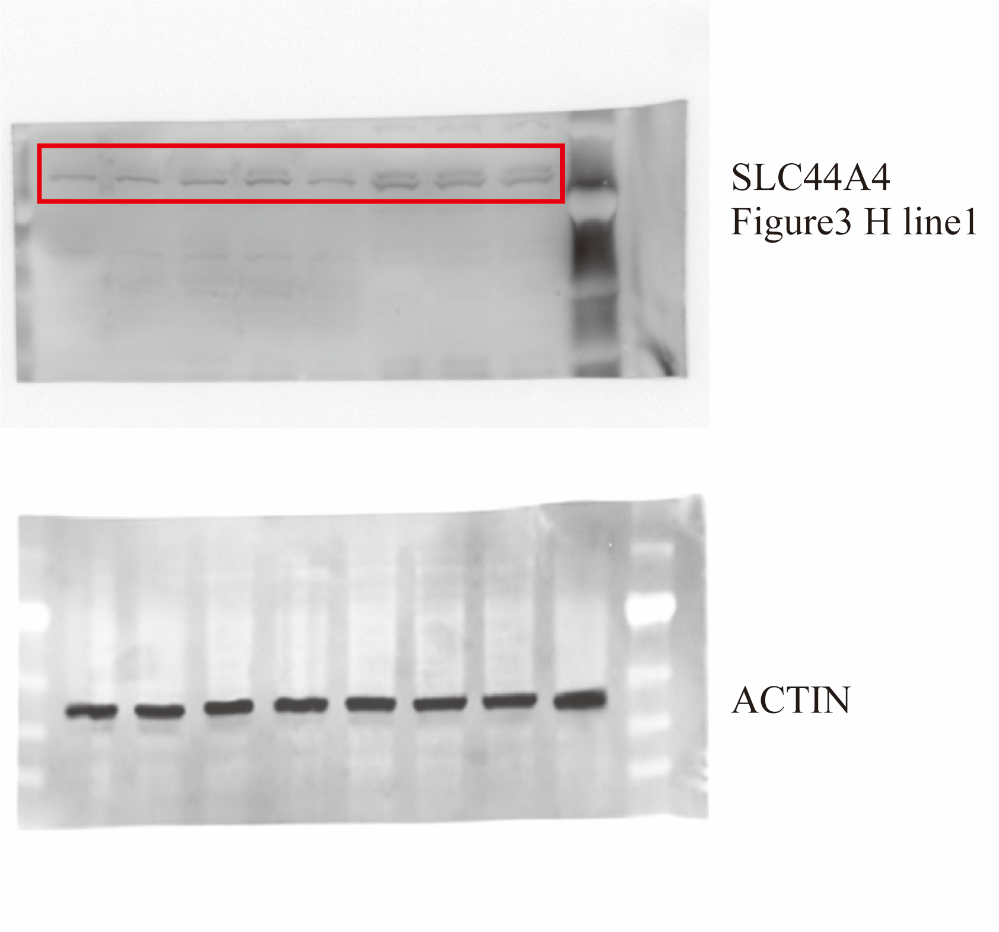

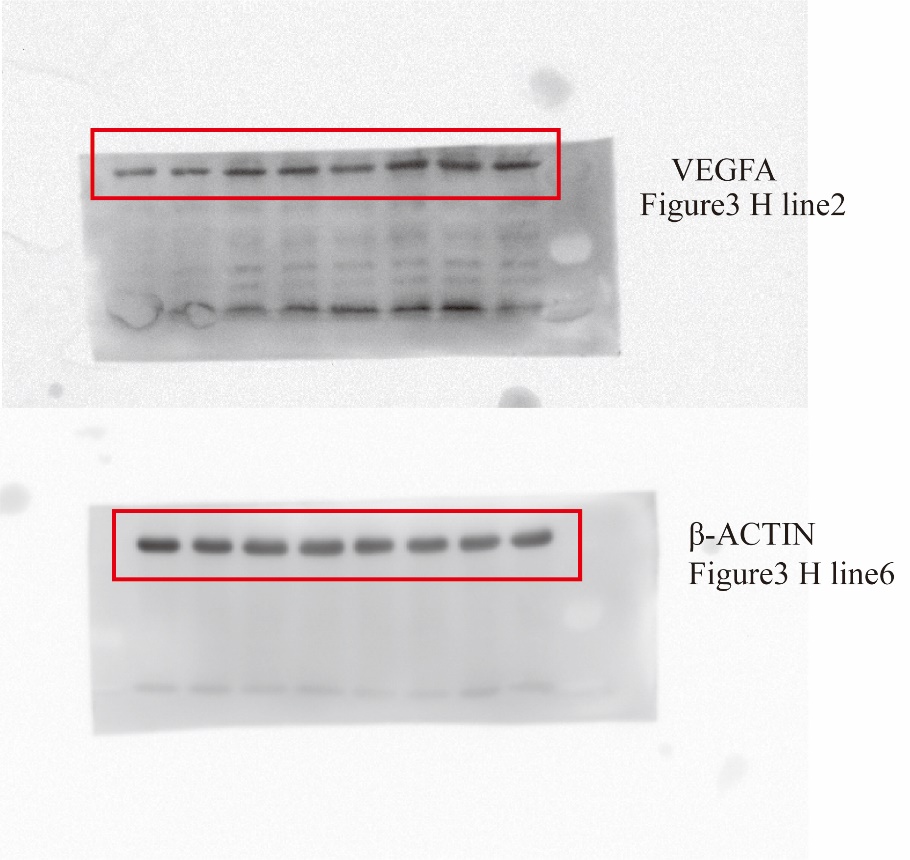

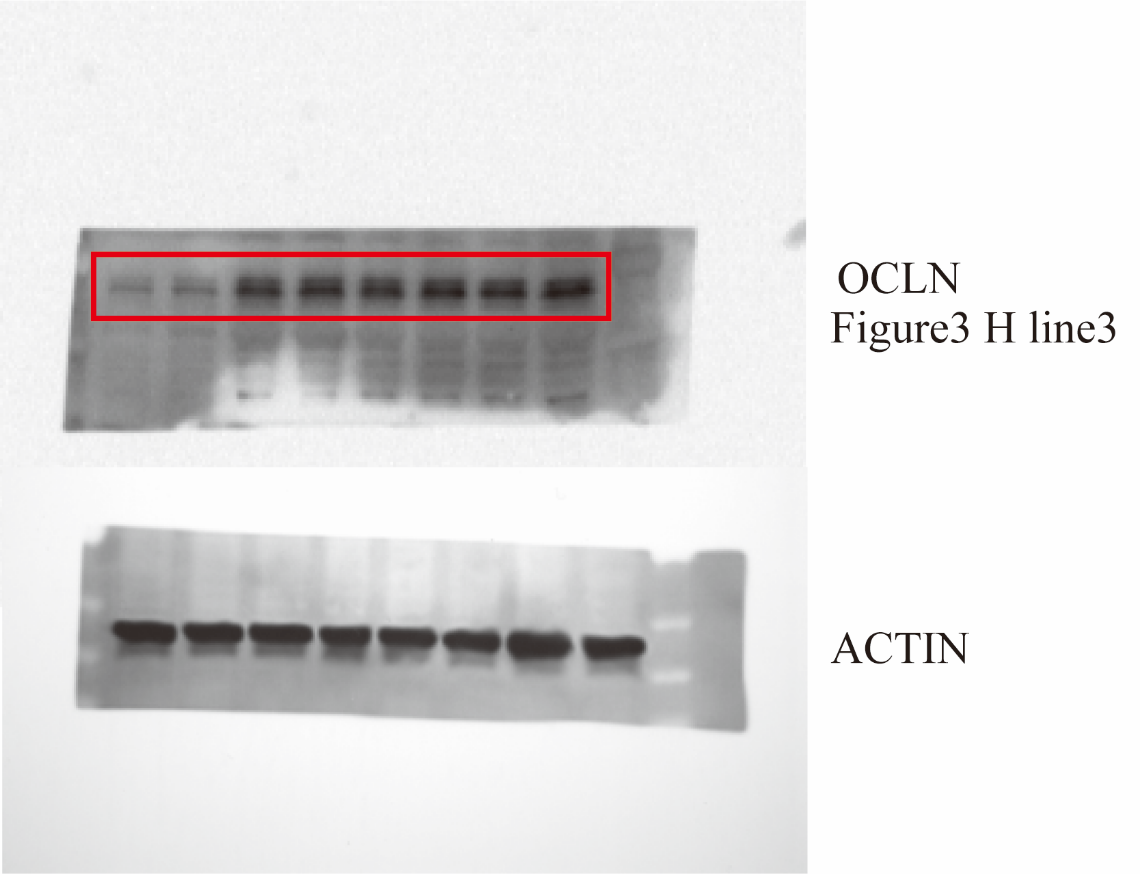

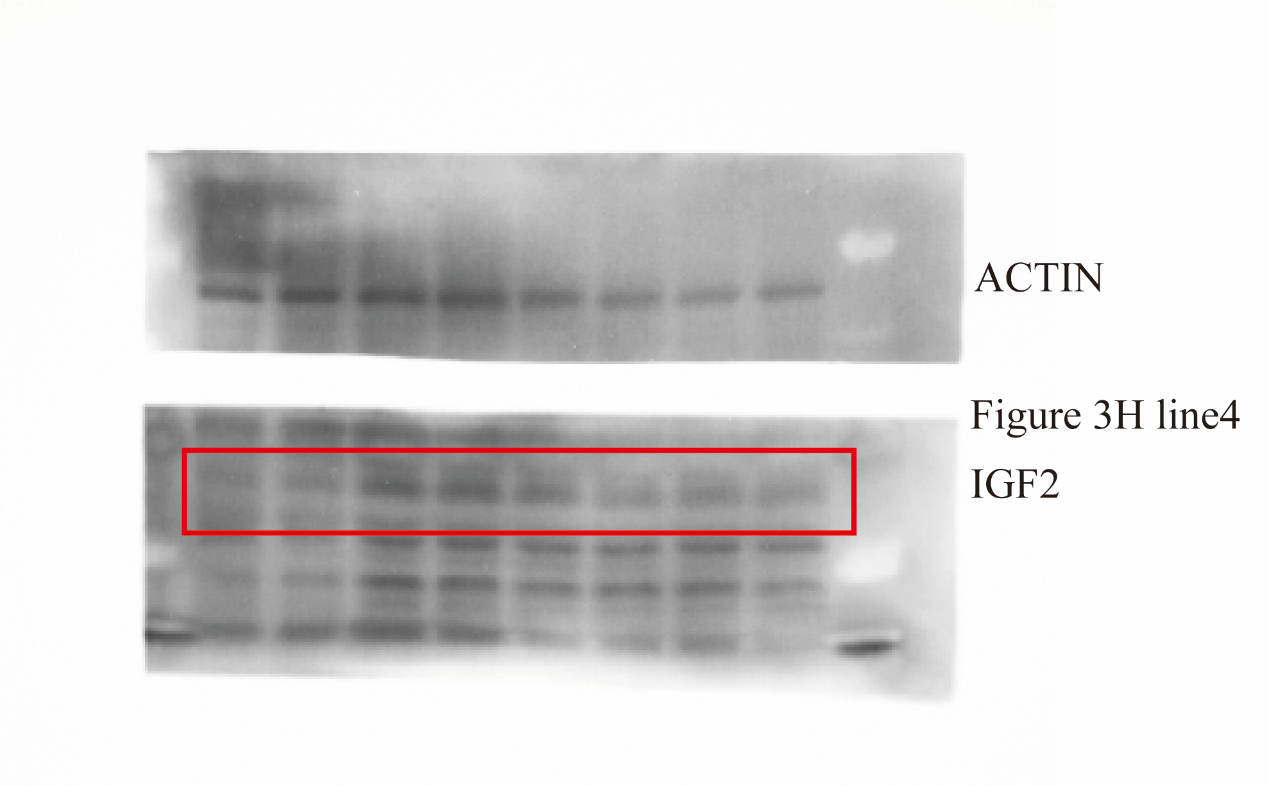

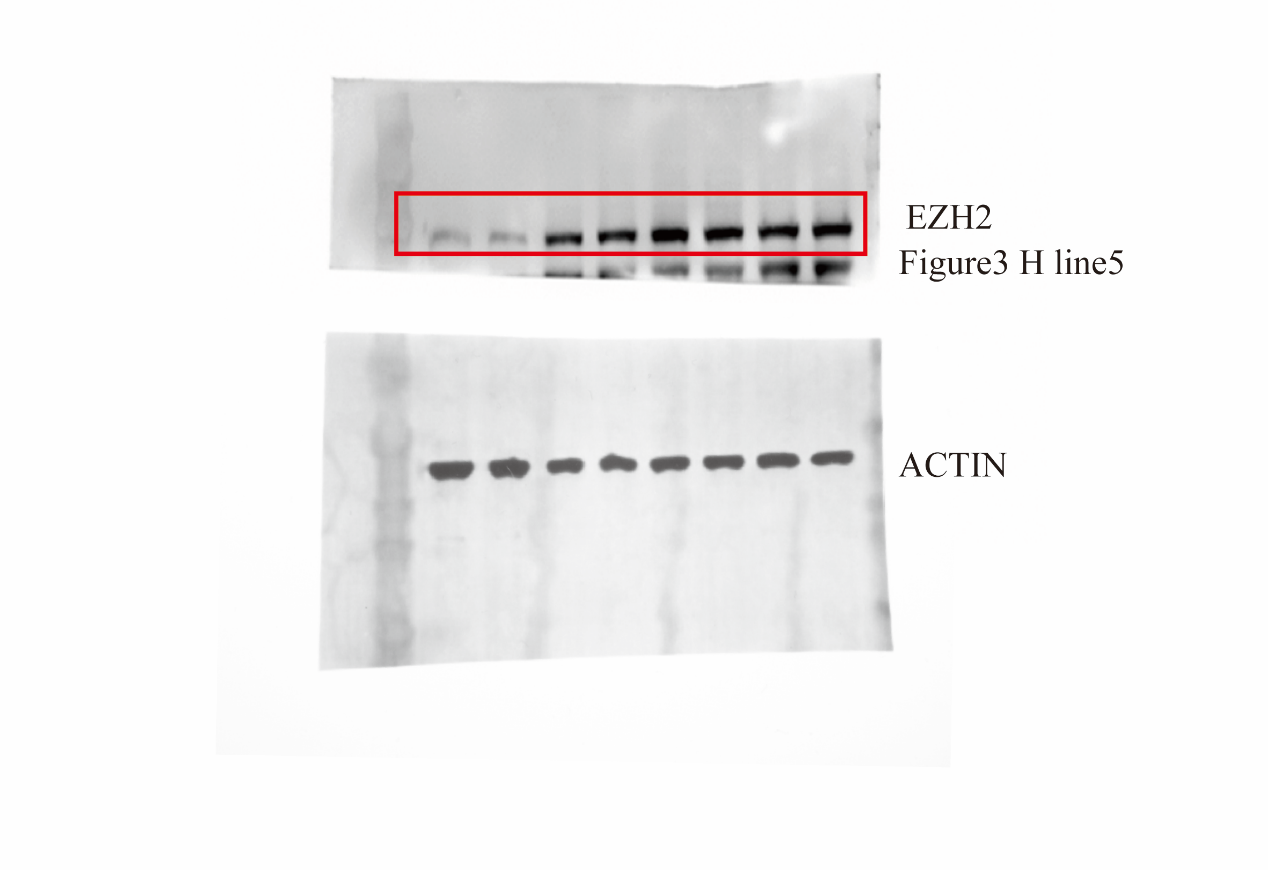


**Figure3 I**


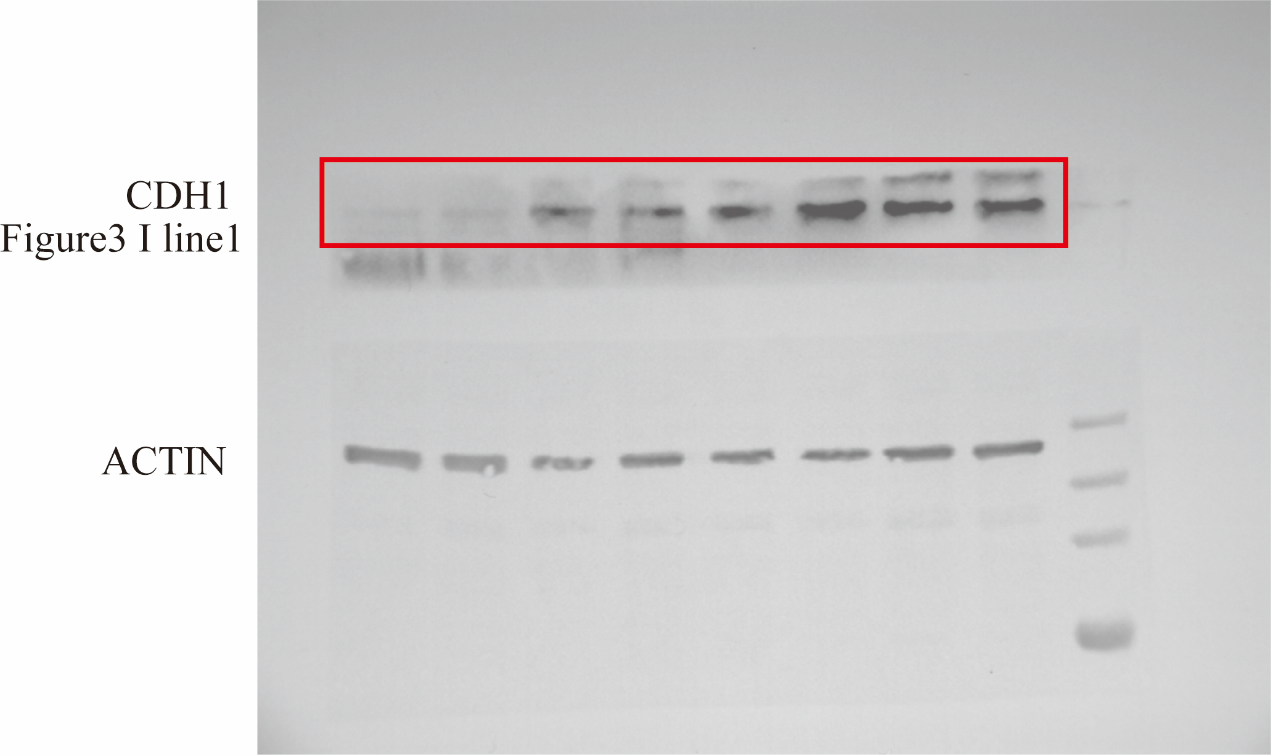


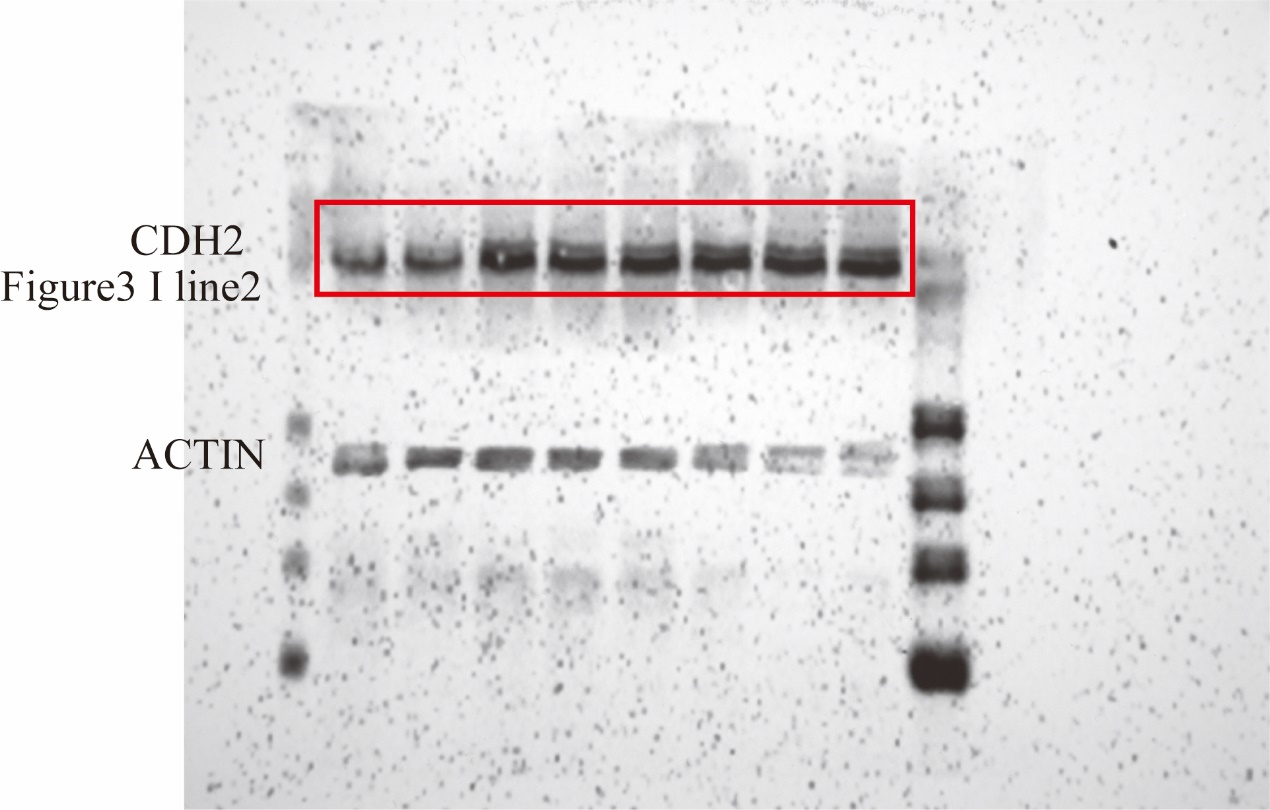

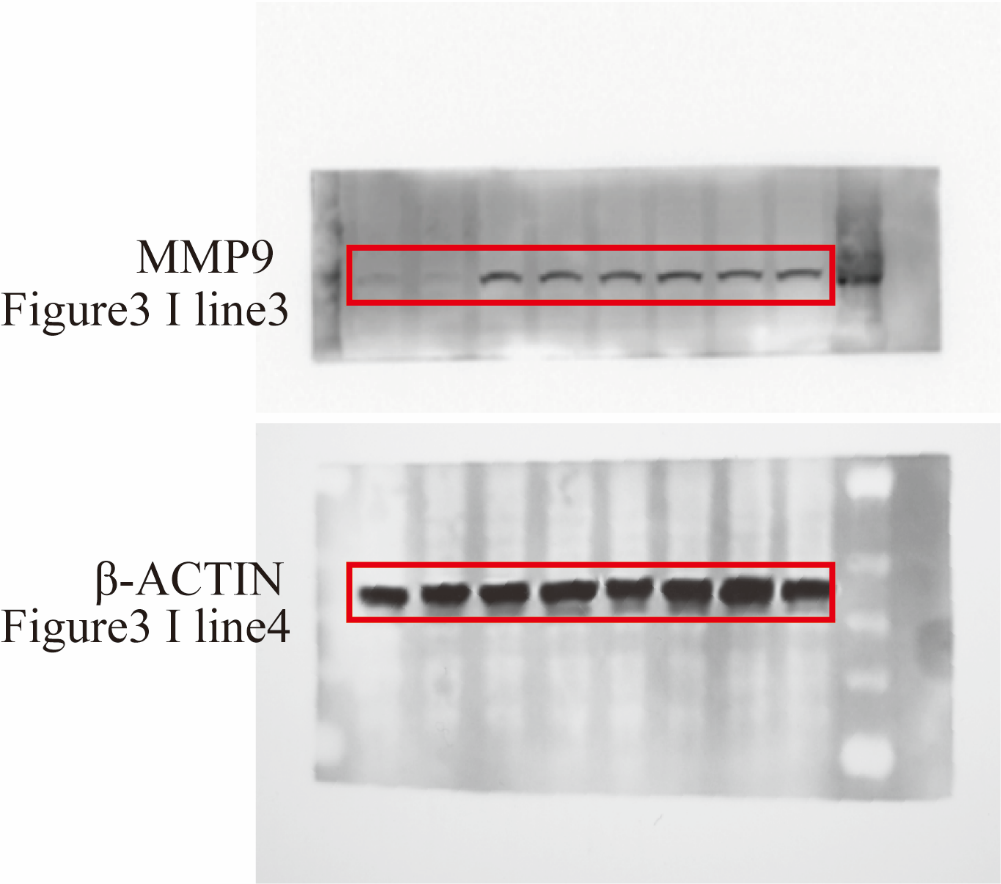

Supplement: Supplementary file 1 — Supplementary Material 1 [file 12885_2024_12244_MOESM1_ESM.docx]
